# Disordered Eating Attitudes and Behavioral and Neuroelectric Indices of Cognitive Flexibility in Individuals with Overweight and Obesity

**DOI:** 10.3390/nu10121902

**Published:** 2018-12-04

**Authors:** Caitlyn G. Edwards, Anne M. Walk, Sharon V. Thompson, Sean P. Mullen, Hannah D. Holscher, Naiman A. Khan

**Affiliations:** 1 Division of Nutritional Sciences, University of Illinois, Urbana, IL 61801, USA; cgedwar2@illinois.edu (C.G.E.); svthomp2@illinois.edu (S.V.T.); hholsche@illinois.edu (H.D.H.); 2 Department of Kinesiology and Community Health, University of Illinois, Urbana, IL 61801, USA; amcclur3@illinois.edu (A.M.W.); spmullen@illinois.edu (S.P.M.); 3 Department of Food Science and Human Nutrition, University of Illinois, Urbana, IL 61801, USA; 4 Neuroscience Program, University of Illinois, Urbana, IL 61801, USA

**Keywords:** feeding behavior, cognition, obesity, event-related potential, P3

## Abstract

Impairment in cognitive flexibility is a trait characteristic among individuals with diagnosed eating disorders. However, the extent to which these relationships exist in individuals with overweight or obesity remains unclear. Furthermore, there is a lack of knowledge characterizing the neural underpinnings of these relationships. The current study aimed to investigate disordered eating attitudes and cognitive flexibility among adults with overweight and obesity. The Eating Attitudes Test (EAT-26) and a task-switching paradigm were collected from 132 adults (50 males, Body Mass Index (BMI) = 32.0 ± 5.8 kg/m^2^). Behavioral measures (accuracy and reaction time (RT)) and neuroelectric indices (amplitude and latency) of the P3 component were assessed. Hierarchical linear regressions, following adjustment of age, sex, intelligence quotient (IQ), weight status, and diet quality were developed using summative and subscale scores of the EAT-26. Higher EAT-26 summative scores, and the Dieting subscale, were related to longer RT. Only the Bulimia and Food Preoccupation subscale was related to longer P3 latency. The relationship between disordered eating attitudes and cognitive flexibility extends to individuals with overweight and obesity and is independent of age, sex, IQ, weight status, and diet quality. These findings are important, as differences in cognitive flexibility can lead to behavioral rigidity. Future work should aim to examine other neuroelectric components to identify where differences driving behavioral latencies may be occurring.

## 1. Introduction

Despite many large-scale public health efforts targeted towards reducing excess energy intake and improving diet quality, many Americans fail to meet federally recommended dietary guidelines [1]. While there are many gaps in current knowledge concerning how to overcome the barriers to improving diet quality, there is limited research on the relationship between the mental or cognitive processes surrounding dietary regulation. Understanding these relationships is increasingly important, as our environment is inundated by aggressively advertised and hedonically rewarding high-fat/high-sugar foods, thus requiring constant control of the drive to consume foods beyond hunger and metabolic needs [2]. 

Exploration of cognitive flexibility, one of many interrelated, yet dissociable, components of cognitive control, may hold particular promise to understanding modulation of eating attitudes and behaviors. Cognitive control allows for the initiation, planning, regulation, and achievement of goal-oriented behavior [3]. Cognitive flexibility, as a component of cognitive control, can be thought of as the ability to appropriately adjust one’s behavior according to a changing environment [4]. In our increasingly obesogenic environment, one where we are constantly barraged by images and marketing strategies aimed at overconsumption, cognitive flexibility becomes increasingly pertinent [5]. For some individuals, particularly those with obesity or disordered eating attitudes, adequately employing cognitive flexibility can be challenging in obesogenic settings that may require choosing less appealing food options in the name of health, in the face of more immediately rewarding options [6,7]. 

Although much work has been dedicated towards behavior change promotion, many individuals perpetually struggle to maintain healthful eating behaviors. Cognitive inflexibility potentially contributes to this lack of success. Previous work has indicated that impairment in cognitive flexibility is a trait characteristic among patients with diagnosed eating disorders [8,9,10]. This research is becoming increasingly relevant as 30 million adults in the United States report suffering from clinically significant disordered eating attitudes at some point in their lives [11]. Infrequently studied is the relationship between disordered eating attitudes and cognitive control among individuals who suffer from sub-clinical disordered eating thought patterns. Indeed, in a lifetime prevalence study of eating disorders, both males and females were more likely to suffer from a “subthreshold binge eating disorder” than either anorexia nervosa or bulimia nervosa [11]. While these subthreshold disordered eating attitudes have been briefly studied, the literature is sparse and particularly lacking in individuals with overweight and obesity [12]. It is possible, and probable, that individuals with these subthreshold disorders will similarly have difficulty adequately regulating their eating behaviors, potentially due to poor cognitive flexibility. 

Cognitive flexibility is commonly assessed using behavioral measures (i.e., accuracy and reaction time (RT)) in task-switching paradigms, but previous literature relating these behavioral measures to disordered eating attitudes lacks proposed mechanisms of action. The use of the electroencephalography and the event-related potential (ERP) technique allows for examination of stimuli response with millisecond precision, allowing for examination of not only the behavioral responses but also the neural underpinnings of said responses. Specifically, the P300 (P3, P3b), a positive-going component occurring roughly 300–700 milliseconds (ms) post-stimulus onset, signifies the resources required for stimulus context updating and resource allocation [13]. In a task-switching paradigm, the P3 is largely thought to reflect how well one can activate currently relevant stimulus-response rules and deactivate previously relevant rules [14]. The P3 amplitude refers to the magnitude to which attentional resources are reconfigured to adapt to the change in stimulus-response rules, while the latency references the speed of this information processing [15]. Captured in these measures is an attentional (changes in stimuli that then require rule-set adaptation) as well as an intentional (changes in the rule-set then requiring selection of motor responses) switch [16]. Examining the P3 component in relation to disordered eating attitudes in a task-switching paradigm may provide novel insights into the potential neural mechanism by which disordered eating attitudes may contribute to poorer cognitive flexibility, or vise-versa.

An additionally important factor to consider when studying the relationship between eating attitudes and cognitive control is weight status. Individuals with overweight or obesity have been previously shown to exhibit poorer performance on cognitive control tasks [17,18]. Given the frequency of co-morbidity between disordered eating attitudes and obesity, poor dietary choices may be a consequence of cognitive flexibility decrements among individuals with overweight and obesity and disordered eating attitudes. This is additionally problematic given the emergence of recent evidence that individuals with a BMI ≥ 25 kg/m^2^ may have greater than two-fold risk for disordered eating [19]. Disordered eating attitudes and behaviors have also been shown to be prevalent across both sexes [11]. However, there is a paucity of data linking disordered eating attitudes to specific aspects of cognitive control, particularly among adults with overweight and obesity. Accordingly, the aim of this study was to elucidate the relationship between eating attitudes and cognitive flexibility in a non-clinical group of men and women with overweight and obesity. We hypothesized that we would observe evidence of disordered eating attitudes in our non-clinical sample. We also hypothesized that higher disordered eating attitudes would be related to lower accuracy and longer RT in a task-switching paradigm. We further aimed to explain these increased RT’s through the examination of the P3 component. We hypothesized that for this component, increased disordered eating attitudes would be related to lower amplitudes and longer latencies. 

## 2. Materials and Methods

### 2.1. Participants and Procedures

Participant characteristics are described in Table 1. Data were collected from 132 adults (50 male), with BMIs ranging from 25.0 to 57.7 kg/m^2^. As an ethnic breakdown of our sample, 1.5% of participants identified as American Indian or Alaskan, 10% identified as of Asian descent, 70% identified as white or Caucasian, 5% identified as Black or African American, and 4% identified as mixed or other. In terms of Social-economic status, 16% of our sample reported an annual household income between 0–$30,000, 56% reported between $30,000–$90,000, and 20% reported greater than $90,000. To qualify for the study, participants had to have a BMI ≥ 25.0 kg/m^2^, be between 25–45 years of age, be free of diagnosed neurological disorders, and free of clinician-diagnosed depression and anxiety disorders. Participants were recruited using flyers posted in community settings, e-mails sent to University employees, as well as word-of-mouth recruitment. To ensure recruitment reached a wide variety of participants, recruitment focused on pursuing individuals outside of the University setting through bus advertisements, postcard mailing in rural neighborhoods, and school flyers in surrounding neighborhoods. Participants were compensated with gift cards upon completion of all study procedures. During the first laboratory visit, participants provided demographic data, completed the EAT-26, as well as height and weight measurements for BMI assessment. The Kaufman Brief Intelligence Test was administered to assess IQ [20]. Participants also completed the National Cancer Institute’s Diet History Questionnaire II to assess overall diet quality [21]. During the second laboratory visit, cognitive testing was conducted following a 10-hour fast in the morning hours to reduce the potentially confounding effects of acute meal consumption on cognitive performance [22]. All participants provided verbal and written consent in accordance with the University of Illinois’ Institutional Review Board and the Declaration of Helsinki.

### 2.2. Intelligence Quotient

KBIT-2 is a test of general intellectual abilities that has been nationally normed for ages 4–90 years [23]. The test is comprised of three subtests: Verbal Knowledge, Riddles, and Matrices. A composite score of the three subtests is then used as a measure of general intellectual abilities.

### 2.3. Habitual Diet Quality

The Healthy Eating Index-2015 (HEI-2015) was calculated using the Dietary History Questionnaire II with portion sizes to assess diet quality. Data were analyzed using National Cancer Institute’s Diet*Calc (Diet*Calc Analysis Program, Version 1.5.0., National Cancer Institute, Epidemiology and Genomics Research Program, Bethesda, MD, USA) software and HEI-2015 scores were generated using HEI-2015 macros in Statistical Analysis System (SAS version 9.4; SAS Institute, Inc., Cary, NC, USA.). HEI scores range from 0–100, based on compliance to 2015–2020 Dietary Guidelines for Americans recommendations. The Healthy Eating Index contains 13 components, including nine adequacy (minimum standard) and four moderation (maximum allowed) components. The component scores were summed to calculate the total HEI score, which was used for analysis. HEI-2015 was calculated to account for the possibility that disordered eating attitudes may influence overall diet quality, which in turn may affect cognitive control processes [24,25].

### 2.4. Anthropometric Measures

Height and weight measurements were completed to calculate BMI (weight (kg)/height (m^2^)). Height and weight were assessed using a stadiometer (model 240; SECA, Hamburg, Germany) and a digital scale (WB-300 Plus; Tanita, Tokyo, Japan). Participant height and weight were assessed while wearing light clothing and no shoes. 

### 2.5. Eating Attitudes Test-26

Disordered eating attitudes were assessed using the self-report 26 question EAT-26 [26]. The EAT-26 has been validated for use in both populations with and without diagnosed eating disorders [27]. The composite EAT-26 score is a continuous variable summative of three subscales: A Dieting subscale, a Bulimia and Food Preoccupation subscale, and an Oral Control subscale. The three subscales tap into different aspects of disordered eating attitudes. The Dieting subscale assesses behaviors related to over concern with weight and calorie content of foods, the Bulimia and Food Preoccupation subscale assesses preoccupations with food and bulimic tendencies, and the Oral Control subscale assesses aspects of self-control [27]. A score on the summative score, as well as the subscales scores, indicates a larger degree of disordered eating attitudes.

### 2.6. Switch Task

**Figure 1:**
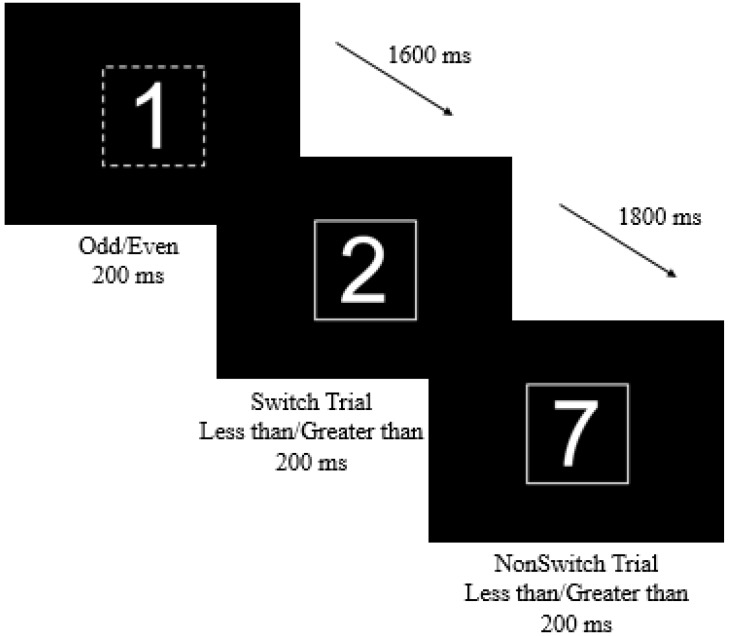
Task stimuli and parameters for the Switch task. Participants completed three task blocks, two homogeneous (within rule-set and dashed/solid boxes) and one heterogeneous (between rule-set and dashed/solid boxes).

Cognitive Flexibility was assessed using a Switch task [14,16]. The Switch task is comprised of three blocks, two homogenous and one heterogeneous. To emulate a real-world scenario where individuals may be forced to switch between rule-sets, the purpose of the homogeneous block is to learn an association, or rule, on certain stimuli (Figure 1). The first homogeneous block is entitled “Less than/greater than” and participants viewed a series of numbers between 1 and 10 inside of a solid box. Participants responded with a right button press if the number was less than 5, and a left button press if the number was greater than 5, thus associating a solid box with the less than/greater than rule-set. The second homogenous block is entitled “Odd/even” and participants viewed a series of numbers between 1 and 10 inside of a dashed box. Participants responded with a right button press if the number was even, and a left button press if the number was odd, thus associating a dashed box with the odd/even rule-set. A test block of 90 trials with jittered inter-stimulus interval (ISI) of either 1600, 1800, or 2000 ms was administered for both homogeneous blocks. In the heterogeneous condition, participants are shown numbers in both solid and dashed boxes. After a practice block of 50 trials, 200 trials of randomized less than/greater than and odd/even stimuli (i.e., randomized homogeneous blocks) were presented and participants were required to switch back and forth between the previously learned mental rule-sets. A Switch trial in the heterogeneous block is defined as a trial in which the previous stimuli belonged to a different mental set than the one presented, or a number was presented inside of a dashed box and followed by a number in a solid box. A NonSwitch trial is defined as a trial in which the previous stimuli belonged to the same mental set as the stimuli presented, or a number was presented inside of a dashed box and followed by another number inside a dashed box. Measures of accuracy and RT for homogeneous and heterogeneous blocks, and for both Switch and NonSwitch trials of the heterogeneous block, were assessed. Global Switch Cost for accuracy was calculated as overall accuracy of the homogenous trials—overall accuracy of the heterogeneous trials, and for RT as overall RT of the heterogeneous trials—overall RT of the homogenous trials.

### 2.7. ERP Assessment

Electroencephalographic (EEG) activity was recorded via a Neuro-scan Quik-cap (Compumedics, Neuroscan, Charlotte, NC, USA) with 64 scalp electrodes arranged in the international 10-10 system. Electrooculographic (EOG) activity was recorded with a set of four electrodes placed at the outer canthus of each eye and above and below the left orbit. A midline sensor placed between Cz and CPz served as a reference and AFz served as the ground. Using a Neuroscan SynampsRT amplifier (Compumedics, Neuroscan, Charlotte, NC, USA), the continuous EEG signal was digitized at a sampling rate of 500 Hz, amplified 500 times to an online low-pass 70-Hz filter with a direct current and a 60-Hz notch filter. Impedance values for all electrodes were maintained ≤10 kohms.

Offline, continuous data were re-referenced to averaged mastoids and merged with behavioral data. An independent components analysis (ICA) was used to systematically reject eye-blink artifacts from the data. Data were submitted to a 0.1-Hz high-pass filter before being submitted to the ICA. ICA and vertical EOG channel correlations greater than 0.35 were considered eye-blinks and were thus rejected. The ICA-corrected EEG data were segmented for each trial beginning −200 ms prior to stimulus onset and continuing 1200 ms post onset. The −200 ms to stimulus onset was used for baseline correction. Data were filtered using a 30-Hz zero phase shift low-pass filter. 

Based on evidence observed from post-hoc topographic images, a 6-sensor region of interest (ROI) comprised of C1, CZ, C2, CPZ, CP1, and CP2 electrodes was used for P3 assessment. Topographic grand average plots were constructed using a stylized topographic map plugin for EEGLAB/ERPLAB [28]. Only correct trials from individuals who reported >50 usable trials in both homogenous and heterogeneous trial types were used for analyses. The P3 component was defined as the localized peak and corresponding latency occurring between 300–600 ms post-stimulus onset. Amplitude was measured as a change score from the pre-stimulus baseline and peak latency was defined as the time point of the maximum amplitude.

### 2.8. Statistical Analysis

**Figure 2:**
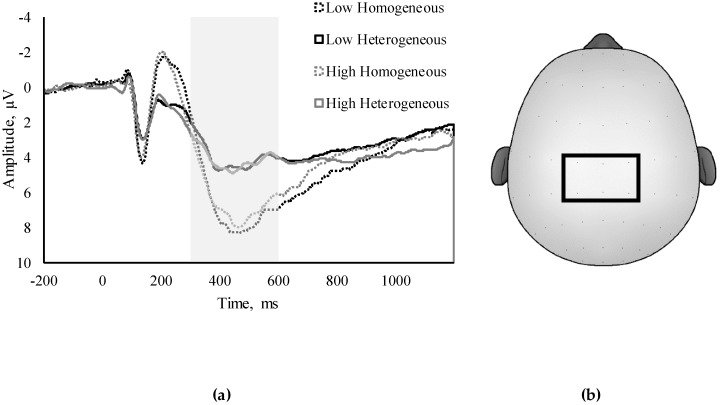
(**a**) Waveform depictions of “low” (*n* = 105) and “high” (*n* = 29) EAT-26 scores of the homogenous and heterogeneous Switch task trials, and (**b**) a topographic representation of the 6 electrode sites (C1, CZ, C2, CPZ, CP1, and CP2) used in the P3 region of interest (ROI).

Statistical analyses were performed with SPSS Statistics version 24 (IBM, Armonk, New York). Internal consistency for the EAT-26 instrument was assessed using Cronbach’s Alpha. Pearson product-moment correlations were conducted to examine bivariate relationships between demographic factors (age, IQ, and sex (females coded as 0 and males coded as 1)), HEI-2015, BMI, EAT-26, and cognitive control variables. Due to non-normality as assessed via Shapiro-Wilk *p* < 0.05, log transformations of all EAT-26 variables were used for all analyses. Hierarchical linear regression analyses were used to determine the contribution of the summative EAT-26 score and each subscale to the cognitive control outcomes. Factors correlated with any one of the cognitive control outcomes were included in step 1 of the regression model. Overall model fit was assessed using ANOVA significance (*p* < 0.05) and tests for multicollinearity were conducted for each subscale. Based on published research demonstrating large effect sizes among clinical samples [9,29], we conducted an a priori power calculation. Specifically, we applied a moderate effect size (*r* = 0.30), two-sided α of 0.05, and 80% power resulting in a minimum sample size target of 81 participants to conduct the multiple regression analyses. For ERP waveform illustrative purposes in Figure 2, participants with “low” (<11) and “high” (>11) EAT-26 responses. An independent *t*-test was conducted among these groups with a significance threshold of *p* < 0.05, and they were plotted for comparison purposes in Figure 2.

## 3. Results

### 3.1. EAT-26

Mean scores from all participants for summative scores and subscales are reported in Table 1. Only 13% of study participants reported a summative EAT-26 score of 0, therefore, 87% of our sample reported some degree of disordered eating attitudes. Three percent of individuals reported scores of 20 or above (the score at which clinician referral is recommended) and 20% of individuals scored above an 11 (the score at which a doctor referral has been proposed for individuals belonging to non-clinical populations) [23,24]. While the EAT-26 score cannot be used to diagnose an eating disorder without also being coupled with an evaluation by a physician, these results indicate that our sample may be largely comprised of individuals who display subthreshold disordered eating attitudes, or disordered eating attitudes at levels that do not qualify for clinical diagnosis. Cronbach’s alpha conducted on the Dieting, Bulimia and Food Preoccupation, and Oral Control subscales yielded scores of 0.79, 0.73, and 0.50, respectively.

### 3.2. Bivariate Correlations

Negative relationships were observed between age and the Bulimia and Food Preoccupation subscale (*r* = −0.26, *p* = 0.003) and between IQ and the Dieting subscale (*r* = −0.18, *p* = 0.04). EAT-26 variables were unrelated to BMI, sex, and HEI-2015 scores (*p* > 0.05).

Regarding the behavioral Switch task outcomes, sex was related to RT of the NonSwitch (*r* = 0.30, *p* = 0.001) and Switch trials (*r* = 0.20, *p* = 0.02), indicating that males took longer to respond on these trials. IQ was related to accuracy of both the NonSwitch (*r* = 0.38, *p* < 0.001) and Switch trials (*r* = 0.30, *p* = 0.001). No relationships were observed between BMI, age, or HEI-2015 scores and behavioral Switch task outcomes of accuracy nor RT. The correlations between EAT-26 variables and Switch task variables are described in Table 2.

Regarding the ERP Switch task outcomes and EAT-26 variables, the EAT-26 summative score was not correlated with any of the ERP variables (all *p’s* > 0.05). The Dieting subscale was positively associated with the Global Switch Cost calculation for accuracy (*r* = 0.17, *p* = 0.05). An inverse trend was also observed between the Dieting subscale and peak P3 amplitude in the homogeneous trials (*r* = −0.17, *p* = 0.06). The Bulimia and Food Preoccupation subscale was negatively correlated with peak P3 latency in the homogenous trials (*r* = −0.23, *p* < 0.01) and trending in its relationships with the NonSwitch (*r* = −0.17, *p* = 0.06) and Switch (*r* = −0.15, *p* = 0.09) trials. The Oral Control subscale was not correlated with any of the ERP variables. In terms of demographic variables, IQ was associated with peak P3 latency in the homogenous trials (*r* = −0.18, *p* = 0.04) and HEI-2015 scores were positively associated with peak P3 amplitude of the Switch trials (*r* = 0.18, *p* = 0.04). Sex, BMI, and age were not correlated with any of the ERP variables. Complete correlations between ERP outcomes and EAT-26 variables are reported in Table 3.

### 3.3. Regression Analyses

Age, sex, BMI, IQ, and HEI-2015 scores were entered into step 1 for each model, as they were shown to be associated with cognitive control outcomes at the bivariate level. Sex was pertinent to the aim of our study, and BMI, age and IQ have been previously associated with cognitive control tasks [3]. For each dependent variable, a standardized step 1 was used, followed by 4 different step 2 regression analyses. The summative EAT-26 and each subscale were therefore entered as separate step 2 variables. Tests for multicollinearity indicated that a low level of multicollinearity was present between the Summative EAT-26 and subscales (variance inflation factor (VIF) Dieting subscale = 1.37, Bulimia and Food Preoccupation subscale = 1.13, Oral Control subscale = 1.31).

Full behavioral regression values are reported in Table 4. For the behavioral Switch task variables, no step 2 variable was associated with accuracy in the homogeneous trials (all *p* > 0.05), indicating that disordered eating attitudes are not associated with accuracy in these trial types. The model for RT in the homogenous trials was significantly improved with the addition of EAT-26 summative score (Δ*R*^2^ = 0.06, *β* = 0.25) as well as with the Dieting subscale (Δ*R*^2^ = 0.06, *β* = 0.26). The addition of the Dieting subscale was associated with an improved model for overall heterogeneous accuracy (Δ*R*^2^ = 0.3, *β* = −0.17) as well as Switch accuracy (Δ*R*^2^ = 0.3, *β* = −0.17) and trending for the NonSwitch accuracy (Δ*R*^2^ = 0.02, *β* = −0.15). These results indicate that individuals with higher Dietary subscale scores took longer to respond on both homogeneous and heterogeneous trial types and performed worse on measures of heterogeneous accuracy. Similarly, the model for RT of the NonSwitch trials was significantly improved with the addition of EAT-26 summative scores (Δ*R*^2^ = 0.04, *β* = 0.20) and with the addition of the Dieting subscale (Δ*R*^2^ = 0.04, *β* = 0.19). RT of the Switch trials was significantly improved following the inclusion of the summative EAT-26 (Δ*R*^2^ = 0.03, *β* = 0.18) and the Dieting subscale (Δ*R*^2^ = 0.04, *β* = 0.20). These results indicate that individuals with higher summative EAT-26 scores took longer to respond on these trial types, and that the Dieting subscale was even further associated with an increased RT, as indicated by the significant Δ*R*^2^. The Dieting subscale was trending with Global Accuracy Cost (Δ*R*^2^ = 0.02, *β* = 0.16), though no relationships were observed for Global RT Cost, nor Local Switch Costs.

The model for latency of the homogeneous P3 trials was significantly improved by the addition of the Bulimia and Food Preoccupation subscale (Δ*R*^2^ = 0.04, *β* = −0.20). Intriguingly, apart from this, no models were predictive of Switch task ERP variables in either the homogenous or heterogeneous task conditions, nor in terms of Global or Local Switch Costs (all *p* > 0.05). Therefore, while relationships were observed between disordered eating attitudes and behavioral accuracy and RT, relationships were largely not seen regarding the P3, indicating that these behavioral decrements may not be driven by differences in P3 amplitude or latency. Waveform depictions are presented in Figure 2.

## 4. Discussion

We aimed to elucidate relationships between disordered eating attitudes and cognitive flexibility in a non-clinical sample of adults with overweight and obesity. As hypothesized, behavioral latencies in a cognitive flexibility task were correlated with higher disordered eating attitudes, specifically the EAT-26 Dieting subscale. Furthermore, the contribution of disordered eating attitudes was generalized such that it was evident for both the homogenous and heterogeneous task conditions. Importantly, this relationship occurred even after controlling for potential confounding factors such as sex, age, BMI, diet quality, and intellectual abilities. However, the only statistically significant relationship observed between measures of disordered eating attitudes and ERP P3 variables was with the Bulimia and Food Preoccupation subscale. These results suggest that the neural mechanisms by which disordered eating attitudes influence cognitive flexibility may be evident without decrements captured by the P3 ERP component, and that future work should examine possible alternative neurophysiological mechanisms of this relationship. 

Our findings are consistent with previous literature indicating that individuals with eating disorders and with overweight or obesity exhibit differential patterns during cognitive flexibility task performance [30,31]. Both homogenous and heterogeneous trial types of the Switch task were related with disordered eating attitudes, as indicated by longer RTs and lower accuracies among individuals with higher Dieting subscale scores. These results also extend previous work relating eating attitudes to cognitive flexibility by demonstrating that cognitive impairment related to disordered eating risk is not limited to clinical populations but is also present among otherwise healthy individuals with overweight and obesity [30]. Interestingly, the relationship between RT and disordered eating attitudes was observed across both the EAT-26 summative score as well as the Dieting subscale for all trial types, suggesting both a generalized and specific negative influence of disordered eating attitudes on processing speed.

While the EAT-26 is a widely used eating disorder screening tool, there is substantially less work evaluating the independent subscales. Evidence in support of the specificity between the Dieting subscale and cognitive flexibility reinforces the theory of functional differences across disordered eating symptoms. The Dieting subscale is thought to reflect “negative body image and avoidance of fattening foods” [32]. Orbitello et al. reported that, in a sample of non-clinical individuals with overweight and obesity, a high Dieting subscale score was a risk factor for eating disorders not otherwise specified (previously EDNOS, now other specified feeding or eating disorder (OSFED)) [27]. Our results reveal that participants with higher Dieting subscale scores took longer to respond in both homogenous and heterogeneous task conditions. Our results indicate that, not only do a large number of participants identify with the Dieting subscale, but also that they exhibit cognitive rigidity patterns similarly evident in clinical studies of patients with eating disorder diagnoses. These results point to a need for more studies examining cognitive barriers to behavior change among community populations.

Intriguingly, in contrast to our hypothesis, the only relationships between EAT-26 variables and P3 outcomes that maintained significance after controlling for pertinent demographic and body composition variables were between the Bulimia and Food Preoccupation subscale and latency in the homogenous task trials. Yet, no relationships were observed between the Bulimia and Food Preoccupation subscale and behavioral variables. This is interesting, as bivariate relationships were observed between the Dieting subscale and the peak amplitude during homogeneous trials, yet this relationship did not persist in regression modeling. To the authors this implies that there may be some relationship between these variables, albeit not very strong. The homogeneous trials are also thought of as the “easier” task trials, in that you are staying within one rule-set, and thus it is intriguing that results were observed here.

The P3 component is largely thought to reflect contextual updating of task-set configurations, or how well one can activate currently relevant stimulus-response rules and deactivate previously relevant rules (in this case the difference prompted by the solid or dashed boxes) [14]. As outlined below, various explanations may explain these relationships. In the present task, motor responses were held constant across conditions, meaning that our task was better able to capture an attentional switch rather than an intentional switch. The lack of associations indicates that decrements in behavioral performance are perhaps not reliant on stimulus evaluation or contextual updating and may instead draw mechanistic foundations from measures of an *intentional* switch, including task-specific motor response remapping, or inhibition of response alternatives. Support for this explanation can also be drawn from the correlations between RT and P3 latency variables. While RT is an index of both duration of stimulus evaluation process as well as response selection, the P3 latency is only reflective of stimulus evaluation [16]. Our results revealed correlations between RT and P3 latency in the homogenous conditions (*r* = −0.31, *p* < 0.01), but no relationships between RT and P3 latency in the Switch and NonSwitch conditions (*r* = 0.12, *p* = 0.18; *r* = 0.07, *p* = 0.43). While interpretations of this result may vary, one can view these relationships as another indication that an individual’s eating attitudes do not have bearing on the stimulus evaluation component of the P3, indicating a need for investigation of disordered eating attitudes using tasks designed to evaluate intentional switches involved in behavior. Another explanation for a lack of a relationship between disordered eating attitudes and P3 outcomes may be that, in this task, while we observed global switch effects we did not observe local switch effects (Figure 1). Further tasks using the ERP technique in conjunction with task-switching paradigms are thus warranted.

While this study draws its strength from the novel use of the ERP technique in conjunction with eating attitude assessment in a large non-clinical sample of individuals with overweight and obesity, our study was not without limitations. Primarily, our study was cross-sectional, and thus any conclusions drawn are correlational, rather than causational. Thus, cognitive flexibility could have been driven by the disordered eating attitudes, or disordered eating attitudes could be maintained due to cognitive rigidity. Further longitudinal work is thus needed to elucidate these relationships. Nevertheless, we were able to shed light on a relatively concordant relationship between eating attitudes and behavioral measures of cognitive flexibility in a non-clinical group of men and women with overweight or obesity. 

## 5. Conclusions

We observed statistically significant relationships between disordered eating attitudes and behavioral RT, yet not neuroelectric indices, on a cognitive flexibility task in a sample of men and women with overweight and obesity. Importantly, these relationships were independent of age, sex, BMI, IQ, and overall dietary quality. More experimental work is necessary to explore the relationship between eating attitudes and cognitive flexibility to determine the cognitive underpinnings of eating behavior regulation and inform future therapeutic approaches to improving adherence to healthful diet habits in the general population with overweight and obesity. 

## Figures and Tables

**Table 1:** Demographic characteristics, weight status, and EAT-26 variables ^1^.

Variable	Group	Female	Male
N	132	82	50
Age, years	33.88 ± 6.00	34.28 ± 5.98	33.22 ± 6.06
IQ	108.48 ± 12.22	107.18 ± 11.67	110.60 ± 12.91
BMI, kg/m ^2^	32.03 ± 5.81 *	33.18 ± 5.39	30.14 ± 6.05
HEI-2015 ^3^	54.33 ± 13.71	53.49 ± 13.56	55.72 ± 13.96
EAT-26 Summative Score	6.73 ± 5.82 *	7.72 ± 7.26	5.12 ± 4.55
Dieting Subscale	4.69 ± 4.38	5.22 ± 4.94	3.82 ± 3.10
Bulimia and Food Preoccupation Subscale	1.45 ± 2.75	1.56 ± 3.20	1.56 ± 3.20
Oral Control Subscale	1.26 ± 1.65	1.37 ± 1.76	1.08 ± 1.44

^1^ Values are means ± standard deviation unless otherwise stated; ^2^ Determined by the Center for Disease Control Body Mass Index (BMI) classifications; ^3^ Calculated using National Cancer Institute Healthy Index (HEI) 2015 Assessment; * Independent *t*-tests revealed significant differences between sex (*p* < 0.05).

**Table 2:** Bivariate Correlations between EAT-26 variables and Switch task behavioral variables.

	Summative EAT-26	Dieting Subscale	Bulimia and Food Preoccupation Subscale	Oral Control Subscale
**Homogeneous**				
Overall Accuracy	−0.09	−0.08	−0.10	0.06
Overall RT	0.25 **	0.26 **	−0.10	0.16
**Heterogeneous**				
NonSwitch Accuracy	−0.17	−0.22 *	0.04	0.03
NonSwitch RT	0.16	0.19 *	−0.12	0.03
Switch Accuracy	−0.19 *	−0.22 **	0.00	−0.02
Switch RT	0.15	0.20 *	−0.04	0.10
**Switch Cost**				
Global Accuracy	0.16	0.21 *	−0.07	0.01
Local Accuracy	0.04	0.03	0.05	0.07
Global RT	−0.01	0.03	−0.02	−0.05
Local RT	0.05	0.10	0.07	0.13

RT—Reaction Time; ** Correlation is significant at the 0.01 level (2-tailed). * Correlation is significant at the 0.05 level (2-tailed).

**Table 3:** Bivariate Correlations between EAT-26 variables and Switch task ERP variables.

	Summative EAT-26	Dieting Subscale	Bulimia and Food Preoccupation Subscale	Oral Control Subscale
**Homogeneous**				
Amplitude	−0.09	−0.17 ^†^	0.05	0.01
Latency	0.01	0.03	−0.23 **	0.11
**Heterogeneous**				
NonSwitch Amplitude	0.08	0.03	0.09	0.04
NonSwitch Latency	0.01	−0.01	−0.17 ^†^	−0.02
Switch Amplitude	0.10	0.06	0.06	0.05
Switch Latency	0.40	0.01	−0.15	0.06
**Switch Cost**				
Global Amplitude	0.13	0.17 *	0.12	−0.01
Local Amplitude	0.00	0.02	−0.09	−0.07
Global Latency	−0.10	−0.08	0.00	−0.16
Local Latency	0.07	0.10	0.04	0.11

** Correlation is significant at the 0.01 level (2-tailed). * Correlation is significant at the 0.05 level (2-tailed). ^†^ Correlation is trending at *p* < 0.07 (2-tailed).

**Table 4:** Hierarchical Regression between pertinent variables and Switch task cognitive outcomes.

	Homogeneous RT			NonSwitch RT			Switch RT		
Step and Variable	*β*	Δ*R*^2^	Model *p*	*β*	Δ*R*^2^	Model *p*	*β*	Δ*R*^2^	Model *p*
Step 1									
Sex	0.07	0.02	0.68	0.30 **	0.14	*p* < 0.01	0.20 *	0.08	0.05
BMI	−0.03			−0.05			−0.11		
IQ	−0.10			−0.12			−0.10		
Age	0.09			0.18 *			0.12		
HEI-2015	0.05			0.02			−0.01		
Step 2									
Summative EAT-26	0.25 **	0.06 **	0.08	0.20 *	0.04 *	*p* < 0.001	0.18*	0.03 *	0.02
Dieting Subscale	0.26 **	0.06 **	0.07	0.20 *	0.04 *	*p* < 0.001	0.20 *	0.04 *	0.01
Bulimia and Food Preoccupation Subscale	−0.08	0.01	0.69	−0.10	0.01	*p* < 0.001	−0.01	0.00	0.09
Oral Control Subscale	0.15	0.02	0.41	0.03	0.00	*p* < 0.001	0.09	0.01	0.06

RT—Reaction Time. ** Correlation is significant at the 0.01 level (2-tailed). * Correlation is significant at the 0.05 level (2-tailed).
